# Clarifications concerning the commentary “Published analysis of contraceptive effectiveness of Daysy and DaysyView app is fatally flawed”

**DOI:** 10.1186/s12978-019-0746-1

**Published:** 2019-06-17

**Authors:** Martin C. Koch, Johannes Lermann, Niels van de Roemer, Simone K. Renner, Stefanie Burghaus, Janina Hackl, Ralf Dittrich, Sven Kehl, Patricia G. Oppelt, Thomas Hildebrandt, Caroline C. Hack, Uwe G. Pöhls, Stefan P. Renner, Falk C. Thiel

**Affiliations:** 1 Frauenklinik, Universitätsklinikum Erlangen, Universitaetsstrasse 21-23, 91054 Erlangen, Germany; 2 Valley Electronics AG, Marienstraße 16, 8003 Zurich, Switzerland; 3 Praxis, Kaiserstraße 26, 97070 Würzburg, Germany; 4 Klinik am Eichert, Frauenklinik, Eichertstraße 3, 73035 Göppingen, Germany

First, we would like to thank the author, Chelsea B. Polis [1], for her input to the discussion about possibilities and risks of fertility awareness based methods (FABMs) and taking her time to extensively reviewing our work [2].

We have gladly read the commentary by Chelsea B. Polis [1] and will try to answer all questions that address the content and the aim of the original study [2] as well as the data analysis.

Within the study, more emphasis was placed on the Pearl-Index (PI) in the abstract and in the discussion compared to the Life-Table Analysis. The main question of the original study was to investigate whether the application of *an additional app* has an influence on the security and use of the fertility awareness-based method (Daysy). In order to have a baseline to which our hypothesis could be tested, reference was made to a previously published work by Freundl et al. Unfortunately, in this older work, only the PI is comparable. Since Daysy is based on the fertility algorithm of Babycomp and Ladycomp from Valley Electronics, it was claimed that Daysy has a similar PI to these products that are ultimately bounded by the fertility awareness-based method itself.

We are aware of the weakness of the PI as a calculation of contraception safety and addressed it accordingly in our manuscript. We agree that a major problem of the PI is that it does not account for the duration of exposure; the PI is reasonably reflective of contraception failure if duration of use is short (i.e. 6 to 12 months) and most users use the method for about this period of time. Therefore, we added (as already approached by the journals peer-reviewers) a life-table analysis. In a life-table analysis (or, in the original study, the equivalent to the Kaplan-Meier approach), a separate failure rate is calculated for each month of use making varying durations of use less problematic. As already published, life-table analysis observed at cycle 13 can roughly be compared to the PI. The Kaplan-Meier approach was used to calculate the overall effectiveness rates. Polis suggests that at least 10 pregnancies occurred to women with fewer than 13 cycles. She does not comment that this subgroup consists of a total of 696 women. Polis further expresses concerns that the investigators did not prospectively collect information regarding perfect or imperfect use of the method for each cycle. We understand this concern, this is why we have already discussed it extensively in the publication itself as a limitation of the study. It is very difficult to carry out a large scale real-life study with a total of 4738 cycles and at the same time have full information about the users. Due to the retrospective nature of the study, it was not possible to determine when and how often users had sexual intercourse on the basis of each cycle. Thus, the calculation of the perfect-use pregnancy rate could only be calculated by analyzing all cycles in which the fertility monitor was correctly used. Thus, we concluded that this could lead to a possible downward bias.

Polis further notes that a survey participation rate of 13% is low. Within the study, 6278 women were contacted, 1969 women (31%) followed the invitation, and 798 women (13%) completed the survey. Compared to similar studies, this response rate is quite high.

In her commentary, Polis notes that the eligibility for participation was limited to registered users with a DaysyView account, but no information is provided on what proportion of overall Daysy users this would represent. The aim of this study was to investigate if the *additional use of an App* would thereby improve the usability of the medical device, and thus possibly enhance the typical-use related as well as the method-related pregnancy rates. As already described above, the method-based algorithm already has an approved PI. For data protection reasons, the authors do not have reproductive access to all Daysy users private contacts. Therefore, it was not possible to recruit an international group using the Daysy fertility tracker exclusively. However, 80% of Daysy users actively use their DaysyView account, so this study represents the largest group. The actual use is likely to be even higher, as many users delete their accounts during pregnancy and start over again after pregnancy.

Polis further advocates that the wording of the questionnaire was confusing for the participants in parts. More precisely, she complains, that the term “unwanted” is not synonymous with “unintended.” First, the original questionnaire was written in German and then translated. This translation was checked by two native speakers for content and correct wording. To check the clarity of the questions, a group of women filled out the questionnaire in advance and gave feedback to the authors. No misinterpretation emerged from this feedback or was adapted accordingly. The authors assume that the definition was clear to all international participants. Secondly, most of the participants (45%) answered the questionnaire in German in which the term “unwanted” is clearly defined.

As Polis writes in her commentary, 64% of Daysy users use other contraceptives in addition to Daysy. She continues to write that this confounding effect is not addressed. In the Life-Table Analysis, this possible effect *was* addressed. In the analysis, a distinction was made between women who only had intercourse on green (infertile days) and women who always used an additional contraceptive. If users had sexual intercourse exclusively on green (infertile) days, the probability of an unwanted pregnancy in fraction is 2.2%, which is slightly higher (n/s) than the incidence of women who have always used an additional contraceptive method (1.92%). The authors know this weakness and, again, have already addressed it in the original publication. The authors conclude that in the digital analysis of temperature data, fertility trackers can reduce the risk of inaccurate input or misinterpretation (as it is done by a computer) of fertility indicators, and they can remind the user that a pregnancy risk exists on red (fertile) days, but they cannot reduce the risk of the additional contraceptive methods or unprotected intercourse. It is a risk of fertility awareness-based method per se. Using fertility awareness-based methods means accepting this risk, as already mentioned in the original publication.

We acknowledge that in her review, the author deals intensively with the quality and effectiveness of fertility awareness-based methods publications and is considering the quality of prospective studies an effectiveness of various FABMs. We request the author to accept that the annotated publication is a retrospective study with all of the known advantages and disadvantages of such. We agree that the reproductive study, like all studies, has the known and named weaknesses and that further, ideally prospective, research is necessary. We believe, as Polis noted in her commentary, that “while some issues in data collection and analysis are not unique to this study,” that the outcome of the study is strong and can be compared with other studies, especially in the field of fertility awareness methods. As a final remark, we would like to point out, that the team of authors attaches great importance to independent and good science. We assume that these values will also be represented by Chelsea B. Polis.

## Editor’s note

The article [2] that the Commentary by Chelsea Polis and this Letter refer to has been retracted [3].
